# Supplementary material for: Cavity-coupled telecom atomic source in silicon
Source: Nat Commun. 2024 Mar 15;15:2350. doi: 10.1038/s41467-024-46643-8 (PMC10943074; doi:10.1038/s41467-024-46643-8)
Supplement: Supplementary file 1 — Supplementary Information [file 41467_2024_46643_MOESM1_ESM.pdf]

Supplementary Information for "Cavity-coupled telecom atomic source in silicon"

Adam Johnston[1,2*], Ulises Felix-Rendon[1,2*], Yu-En Wong[1,2*], Songtao Chen[1,3†]

[1] *Department of Electrical and Computer Engineering, Rice University, Houston, TX 77005, USA*
[2] *Applied Physics Graduate Program, Smalley-Curl Institute, Rice University, Houston, TX 77005, USA*
[3] *Smalley-Curl Institute, Rice University, Houston, TX 77005, USA*

1 Experimental configuration

1.1 Detailed experimental setup

In this section we give a more detailed description of our experimental setup, shown in Fig. S1. The fiber-coupled telecom CW tunable laser (Toptica CTL 1320) is frequency stabilized via a wavemeter (Bristol Instruments 871A) with a repeatability of 1.7 MHz. The analog error signal generated from the wavemeter is fed into the laser to perform PID-enabled laser locking. Excitation pulses are generated through a series of acousto-optic modulators (AOMs) and an electro-optic modulator (EOM). The AOM set consists of two fast-switching (10 ns speed) fiber AOMs (AeroDiode 1310-AOM-2) and one slow-switching free-space AOM (ISOMET M1205-P80L-0.6), providing a total extinction ratio of 178 dB. The intensity-modulated EOM is driven with a RF signal to generate laser sidebands. The generated laser pulses pass through a series of neutral density (ND) filters to control the optical power before entering the fiber network towards the devices.

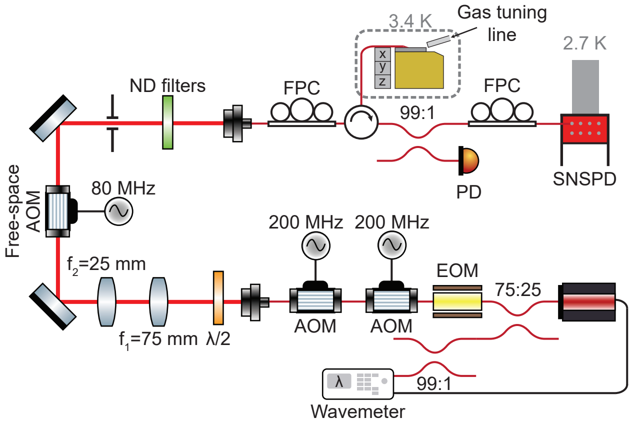

Fig. S1. **Experimental setup.** A more detailed version of the experimental setup shown in Fig. 2a of the main text.

The laser pulses pass through a polarization insensitive fiber circulator (Newport F-CIR-13-P-FP, insertion loss = 0.45 dB) before reaching the devices via the angle-polished fiber. The fiber is polished using an automatic fiber polishing machine (ULTRA TEC, NANOpol) at an angle of 37.97° for optimal coupling with the GC. The device is situated on the cold finger of a closed-cycle cryostat (Montana Cryostation s50) at a temperature of $T = 3.4$ K. The cryostat has a magneto-optical component, which can apply a single-axis magnetic field up to 400 mT. The angle-polished fiber is mounted on a three-axis nanopositioner (Attocube ANPx101/LT, ANPz102/LT) to optimize its coupling to the GC. The T center emission signal is routed by the circulator and sent into the superconducting nanowire single photon detector (SNSPD, Single Quantum EOS 210CS), which operates at a base temperature of $T = 2.7$ K. A small fraction of the emission signal is sent to an InGaAs photodiode (PD, Newport 2053-FC) for monitoring the cavity spectrum. The SNSPD is biased using a waveform generator (Rigol DG832), and the voltage pulses from the SNSPD are counted using a time tagger (Swabian Instruments, Time Tagger 20).

*These authors contributed equally to this work
†songtao.chen@rice.edu

1.2 Cavity tuning

The cavity resonance blue shifts about 9.8 nm when the device is cooled down from the room temperature to $T = 3.4$ K. Gas tuning [1] is implemented to tune the PC cavity resonance to match with the single T center optical transition. We supply clean dry N_2 gas through a gas line inside the cryostat pointing to the device. Nitrogen ice then forms on the device surface, causing a red shift of the cavity resonance up to about 5 nm (850 GHz). To increase the tuning accuracy, we realize blue tuning of the cavity via optical pulses with high optical power to desorb the nitrogen ice. We send a 50% duty cycle pulse train consisting of 1 μs wide optical pulses with 14.61 μW power at $\lambda_0 = 1326$ nm to the cavity. Depending on the number of pulses, we can fine control the amount of blue tuning (Fig. S2). The blue tuning process has an tuning accuracy $\sim$ 0.1 GHz. The condensation of the background gas causes a constant red shift of the cavity resonance at a rate of $\sim$ 10 MHz/min. We apply blue tuning in between experiments to compensate the background drift.

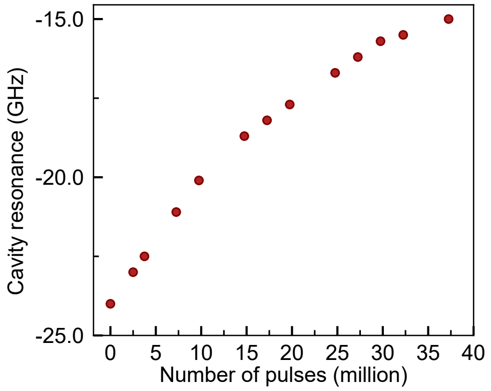

Fig. S2. **Blue tuning of the cavity resonance.** The cavity resonance frequency is blue tuned by sending in optical pulses. The pulses are generated and controlled the same way as in the main experiments.

1.3 Photon collection efficiency

The system photon collection efficiency (η_{sys}), which represents the probability of a single photon emitted to the cavity being registered by the detector, is determined by $\eta_{\text{sys}} = \eta_{\text{cav}}\eta_{\text{GC}}\eta_{\text{path}}\eta_{\text{SNSPD}}$. Here $\eta_{\text{cav}} = \kappa_{\text{wg}}/(\kappa_{\text{wg}} + \kappa_{\text{sc}})$ is the fraction of the photons in the cavity that couple to the waveguide, with κ_{wg} and κ_{sc} as the waveguide and scattering loss channels of the cavity, respectively; η_{GC} is the one-way GC coupling efficiency at $\lambda_0 = 1326$ nm; η_{path} is the transmission of the optical fiber network between the angle-polished fiber and the SNSPD; and η_{SNSPD} is the SNSPD detection efficiency. A cavity efficiency $\eta_{\text{cav}} = 0.358$ is estimated by measuring the ratio (R) of cavity reflection level on- and off-resonance using the relation $R = (1 - 2\eta_{\text{cav}})^2$ and assuming the cavity is under-coupled. We thus can determine the system photon collection efficiency to be $\eta_{\text{sys}} = 0.091$ for the device that contains the single T center in the cavity ($\eta_{\text{GC}} = 0.461$, $\eta_{\text{path}} = 0.786$, $\eta_{\text{SNSPD}} = 0.703$). For the cavity-coupled single T center analyzed in the main text, we observe an excited state Purcell factor $P_t = 5.88$ when the cavity is on-resonance. The probability of the T center decaying into the resonant cavity mode is given by $\beta = P_t/(P_t + 1) = 0.855$.

Due to technical difficulties, there exists a finite time window of $t_0 = 170$ ns in which the fluorescence from the single T center cannot be collected. Depending on the single T center lifetime τ_{exc}, the amount of T center fluorescence that can be collected will be determined as $e^{-t_0/\tau_{\text{exc}}}$. When the cavity-coupled single T center emission is saturated, which corresponds to an incoherent excitation probability of 0.5, we are expecting a count level of $0.5\eta_{\text{sys}}\beta e^{-t_0/\tau_{\text{exc}}} = 0.011$. This prediction matches with the observed saturation count level of 0.01 photon per excitation pulse.

2 Silicon photonic device design and fabrication

2.1 Cavity design and Purcell factor

The PC cavity used in the experiments consists of an 1D array of elliptical holes, with the defect formed by parabolically decreasing the lattice constant in the center of the cavity [2]. The PC cavity design is carried out using the photonic calculation packages MPB [3] and MEEP [4]. The device layer thickness is $d_z = 220$ nm, and we use refractive indices for silicon and silicon dioxide of $n_{\text{Si}} = 3.505$ and $n_{SiO_2} = 1.466$, respectively. By

maximizing the photonic bandgap while ensuring the correct symmetry, we obtain the final geometric parameters $(w, h_x, h_y, a_{\mathrm{mir}}, a_{\mathrm{cav}}) = (410, 140, 203, 334, 282)$ nm, where w is the beam width, h_x and h_y are the two major axes of each PC hole, a_{mir} and a_{cav} are the lattice constant for reflector and cavity center regions, respectively. The simulated cavity field distribution is shown in Fig. S3 with $N_{\mathrm{cav}} = 14$ cavity holes at the center and $N_{\mathrm{mir}} = 4$ mirror holes on each side. The mode volume of the cavity is calculated as $V_{\mathrm{mode}} = 0.031\ \mu\mathrm{m}^3 = 0.172(\lambda_0/n_{\mathrm{eff}})^3$. To make an effective one-sided cavity for reflection measurements, we add 10 more mirror holes at the left end of the cavity.

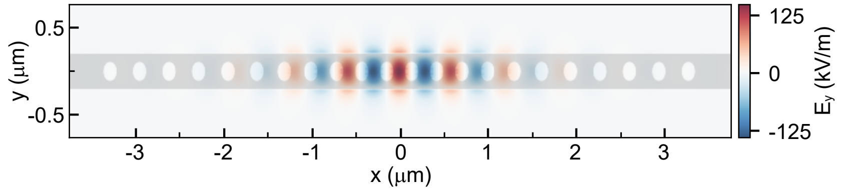

Fig. S3. **Cavity Mode profile.** Top view of the E_y component of the electrical field in the cavity, at a z-cut in the middle of the silicon device layer ($z = 110$ nm).

The Purcell factor for the T center zero phonon line (ZPL) transition can be calculated as $P_{\mathrm{ZPL}}^{\mathrm{sim}} = 4g(\mathbf{r})^2/(\tilde{\kappa}\Gamma_0)$, where $\Gamma_0 = 2\pi \times 169.3$ kHz is the spontaneous emission rate [5], $g(\mathbf{r}) = |\mathbf{d}_{\mathrm{ZPL}}\mathbf{E}(\mathbf{r})|/\hbar$ is the local coupling constant, and $\tilde{\kappa}/2\pi = 7.11$ GHz is the linewidth determined in the detuning-dependent cavity enhancement (Fig. 4b of the main text). The ZPL transition dipole moment $\mathbf{d}_{\mathrm{ZPL}}$ can be determined using the following equation, including a local field correction [6],

$$(\mathrm{DW}\eta_{\mathrm{QE}})\Gamma_0 = \gamma_{\mathrm{ZPL}} = \left(\frac{3n_{\mathrm{Si}}^2}{2n_{\mathrm{Si}}^2 + 1}\right)^2 \frac{n_{\mathrm{Si}}\mathbf{d}_{\mathrm{ZPL}}^2\omega^3}{3\pi\epsilon_0\hbar c^3}, \tag{S1}$$

where $\mathrm{DW} = 0.23$ is the Debye-Waller factor for the T center [5] and $\omega = 2\pi \times 226141.974$ GHz is the cavity-coupled single T center optical transition frequency. Depending on the quantum efficiency η_{QE}, we will have different $\mathbf{d}_{\mathrm{ZPL}}$ (Fig. S4a). In an ideal case of $\eta_{\mathrm{QE}} = 1$, the ZPL dipole moment will be $|\mathbf{d}_{\mathrm{ZPL}}| = 1.67 \times 10^{-30}$ C-m (0.50 Debye). The predicted ZPL Purcell factor distribution inside the silicon cavity are shown in Fig. S4b and Fig. S4c. At the cavity center, assuming perfect dipole alignment with the cavity polarization, we will have the maximum Purcell factor $P_{\mathrm{ZPL}}^{\mathrm{sim}} = 470$ and the coupling $g = 2\pi \times 376$ MHz. From the measured cavity-enhanced decay, we can also predict the $P_{\mathrm{ZPL}} = P_t/(\mathrm{DW}\eta_{\mathrm{QE}})$. Due to the suboptimal positioning of the single T center inside the cavity and imperfect dipole alignment with the local cavity polarization, we will have $P_{\mathrm{ZPL}} \leq P_{\mathrm{ZPL}}^{\mathrm{sim}}$. This enables us to extract a lower bound of the quantum efficiency of $\eta_{\mathrm{QE}} \geq 23.4\%$ (Fig. S4d).

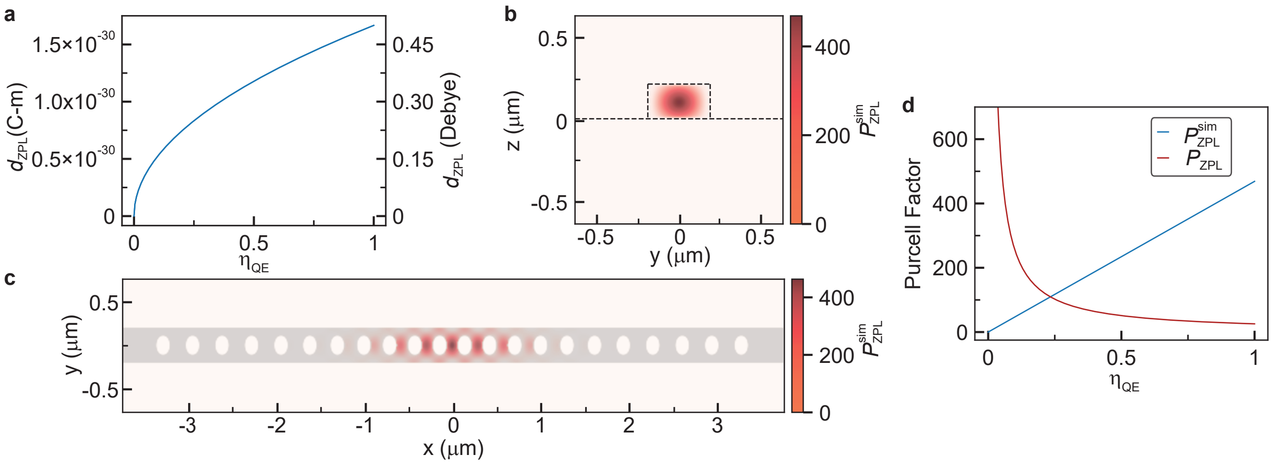

Fig. S4. **T center ZPL dipole moment and Purcell factor. a.** Dependence of T center ZPL transition dipole moment on the η_{QE}. **b.** Simulated Purcell factor distribution in the yz-plane (at $x = 0$) assuming $\eta_{\mathrm{QE}} = 1$. **c.** Similar plot as panel **b** but now in the xy-plane (at $z = 110$ nm). **d.** Simulated and indirectly measured ZPL Purcell factor assuming different η_{QE}.

2.2 Grating coupler design

The light is coupled into and out-of the devices via a 2D subwavelength GC, shown in Fig. 1b of the main text. The GC consists of 20 periods of fully-etched PC holes with a triangular lattice [7, 8], and a width set to 13.1 μm to maximize the mode overlap along the y-direction between the GC diffracted light and a SMF-28 fiber (mode field diameter = 9.2 μm). A uniform grating structure does not provide a good mode overlap with the fiber Gaussian mode [9]. Instead we turn to an apodized structure to fine tune the diffraction strength at each period to maximize the mode overlap.

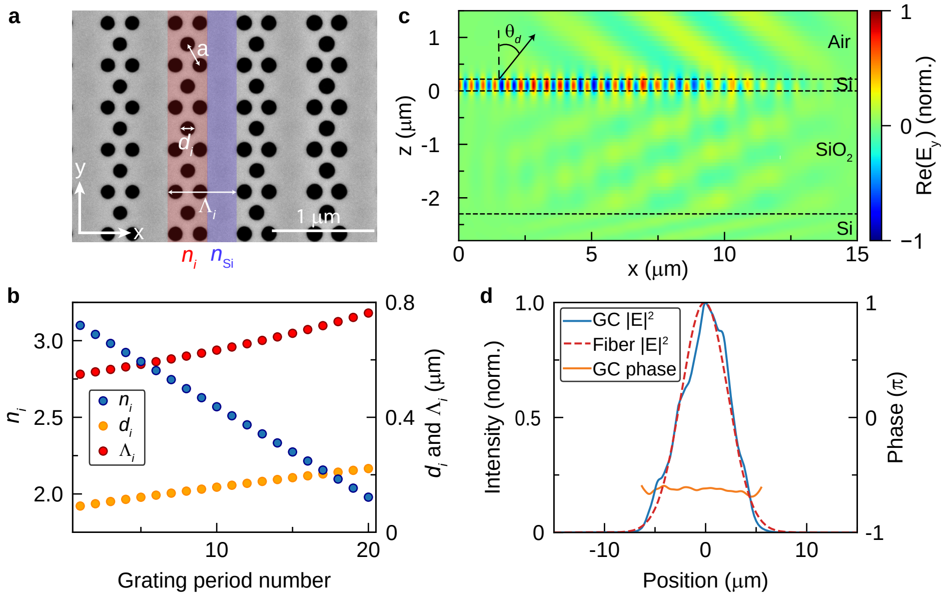

Fig. S5. **Subwavelength grating coupler design. a.** Scanning electron microscope image of a section of the GC. Structural parameters of the GC are labeled on the image. Here, n_i, Λ_i, d_i and a are the effective index, period, PC hole diameter and PC hole pitch in each grating period, respectively. The duty cycle of the grating structure is kept constant at $1-(a+d_i)/\Lambda_i = 0.4$. **b.** Parameters of n_i, Λ_i and d_i for the optimized apodized grating structure. **c.** Simulated electric field (E_y) radiated by the GC in the xz-plane (at $y = 0$). θ_d denotes the diffraction angle. **d.** Simulated intensity and phase profiles of the GC diffracted field along $(-\cos(\theta_d)\hat{x} + \sin(\theta_d)\hat{z})$ direction, and the mode profile (red dashed line) of a SMF-28 fiber.

Three-dimensional Lumerical (Ansys) finite-difference time-domain (FDTD) simulations are used to optimize the coupling efficiency of the GC, $\eta_{\text{GC}} = D \times O^x_{\text{GC}} O^y_{\text{GC}}$ at $\lambda_0 = 1326$nm, where D is the directionality of the GC, quantifying the ratio of the light diffracted towards the fiber; O^x_{GC} and O^y_{GC} are the mode overlap between GC diffracted light and the fiber mode along $(-\cos(\theta_d)\hat{x} + \sin(\theta_d)\hat{z})$ and $\hat{y}$ directions, respectively. To improve the mode overlap O^x_{GC}, the effective index n_i (Fig. S5a) for each grating period is linearly apodized by changing the diameter (d_i) of the PC holes [8] while keeping the lattice pitch constant ($a = 239$ nm). The period Λ_i is adjusted accordingly to ensure a constant diffraction angle θ_d at different grating periods. To fully utilize the SOI structure, the thickness of the buried oxide layer is customized as $d_{\text{BOX}} = 2.3$ μm to create constructive interference in the GC diffraction and thus increase the directionality D. The detailed procedure for optimizing the GC efficiency can be found in Ref. [10, 11].

The optimized geometric parameters are summarized in Fig. S5b, where the initial grating period and effective index are $\Lambda_1 = 550$ nm and $n_1 = 3.1$, respectively. The index change between neighbouring period is $\Delta n = 0.059$. The electric field distribution of the GC diffracted field is shown in Fig. S5c with a diffraction angle $\theta_d = 20.88°$ and a directionality $D = 76.3\%$. The fiber polishing angle can be calculated as $\alpha = [90° - \sin^{-1}(\sin\theta_d/n_{\text{fiber}})]/2 = 37.97°$ ($n_{\text{fiber}} = 1.4676$). The mode overlap between the GC diffracted field and the fiber Gaussian mode are $O^x_{\text{GC}} = 98.6\%$ (Fig. S5d) and $O^y_{\text{GC}} = 98.9\%$. After taking into account the transmission of the linearly tapered waveguide (95.9%) and the glass-air interface reflection loss (3.6%), we calculate a maximum one-way coupling efficiency $\eta_{\text{GC}} = 68.8\%$.

During fabrication, we add a constant change on all the PC hole sizes in the GC to tune the GC spectrum center to about 1331 nm at room temperature. After cooling down, we observe ~ 5 nm blue shift for the GC spectrum. The typical GC efficiency at room temperature is $\eta_{\text{GC}} \sim 60\%$ at the GC spectrum center in a measurement setup with full six degrees of freedom (x, y, z, pitch, yaw, rotation) control of the fiber. After fiber gluing (Stycast 2850FT, Catalyst 9) and mounting onto the three-axis (x, y, z) piezo nanopositioner, η_{GC} typically drops by $\sim 5\%$, which is likely due to the change of the pitch angle of the fiber with respect to the sample.

2.3 Nanofabrication process

Our nanofabrication process uses electron beam lithography and reactive ion etching to define nanophotonic structures on the SOI samples (Fig. S6). The detailed process can be found in the Methods section of the main text.

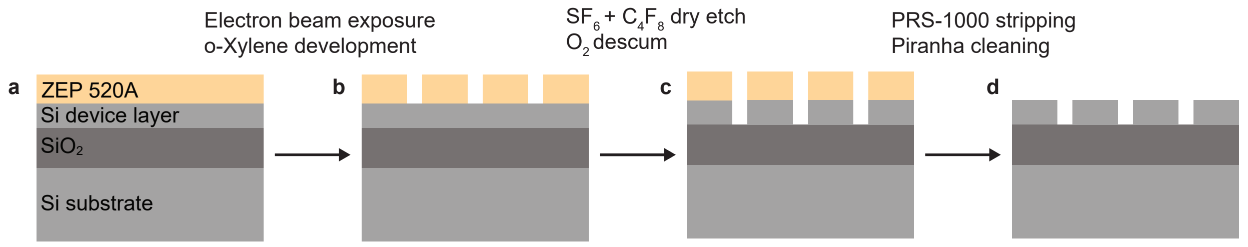

Fig. S6. **Nanofabrication process flow. a.** Cross-sectional view of a SOI sample with an electron beam resist layer on top. **b.** The nanophotonic circuit pattern forms on the resist layer after electron beam writing and development. **c.** Reactive ion etching transfers the pattern to the silicon device layer, followed by oxygen descum. **d.** Resist stripping and piranha cleaning.

2.4 Cavity quality factor vs. T center generation

To determine the potential influence of the T center generation process on the cavity quality factor, we fabricate devices on both implanted and unimplanted SOI samples. The implanted sample has an equal fluence of 5×10^{13} cm^{-2} for ^{12}C and ^{1}H implantation and has gone through corresponding thermal annealing steps, following the procedure discussed in Section 3.1. The two samples' respective ebeam writings are done under the same exposure condition using the same pattern. The ebeam writing tool's conditions (e.g., beam focus, stigmation, beam current) have been kept the same for the two writings to the best of our control. The two samples are then placed on the same carrier wafer and etched simultaneously during the etching step.

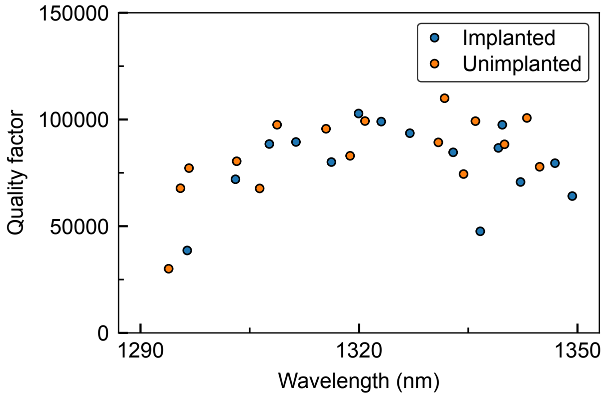

Fig. S7. **Cavity Quality factors.** Measured cavity quality factors (Q) for devices fabricated on the implanted and the unimplanted samples. No significant difference in the device Q performance is observed between the two samples.

To ensure finding cavities that can be gas tuned into resonance with the T center optical transitions, we sweep the hole sizes in the PC cavities, which changes the cavity resonance frequency. The measured quality factors for the two samples are shown in Fig. S7, revealing $Q = (8.0 \pm 1.8) \times 10^4$ and $Q = (8.4 \pm 1.8) \times 10^4$ for implanted and unimplanted SOI samples, respectively. This confirms that the T center generation process has a minimal effect on the cavity quality factor up to an implantation fluence of 5×10^{13} cm^{-2}.

3 T center generation via ion implantation

3.1 Generation protocol

Secondary ion mass spectroscopy (SIMS, Eurofins EAG) analysis of our SOI device layer before ion implantation shows that the native carbon and hydrogen densities are $[\mathrm{C}] = 3 \times 10^{17}$ cm^{-3} and $[\mathrm{H}] \approx 5 \times 10^{17}$ cm^{-3}, respectively. We note that the hydrogen density is close to the SIMS tool limit for hydrogen species (1×10^{17} cm^{-3}).

We use the stopping and range of ions in matter (SRIM) software package [12] to estimate the ion implantation energy necessary for the carbon and hydrogen ions to end up in the middle of the SOI device layer ($d_{\mathrm{depth}} = 110$ nm). We determine the implantation energy for ^{12}C and ^{1}H to be 35 keV and 8 keV, respectively, for an ion implantation direction of 7° from the normal incidence to avoid channeling effect in silicon [13]. With an equal fluence of 1×10^{14} cm^{-2} for both ^{12}C and ^{1}H, the simulation suggests a peak density of 1×10^{19} cm^{-3} for both species in the middle of the device layer (Fig. S8a). The FWHM linewidth of the distribution is 89.3 ± 0.8 nm and 79.8 ± 0.6 nm for ^{12}C and ^{1}H implantation, respectively. To verify this, we implement sequential ion implantation test runs for ^{12}C and ^{1}H (with equal fluence of 1×10^{14} cm^{-2}) without any thermal annealing process. The SIMS measurement of this test sample (Fig. S8b) matches well with the SRIM simulation results.

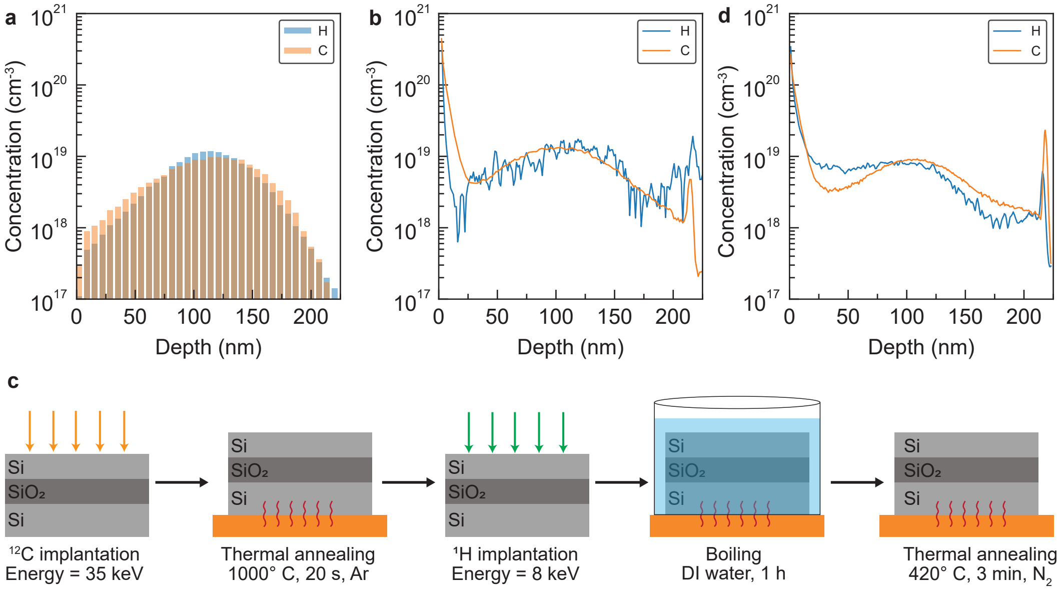

Fig. S8. **T center generation process a.** SRIM simulation results for 35 keV ^{12}C and 8 keV ^{1}H implantation with an equal fluence of 1×10^{14} cm^{-2}. **b.** SIMS analysis of a test sample that goes through the same implantation conditions simulated in panel **a**. No thermal annealing process is performed for this test sample. **c.** Process flow for T center generation. **d.** SIMS analysis of a SOI sample that goes through the T center generation process. The implantation fluence is 1×10^{14} cm^{-2} for both ^{12}C and ^{1}H.

The procedure to generate T centers is shown in Fig. S8c, and detailed in the Methods section of the main text. We implant a SOI sample following the procedure with an equal fluence of 1×10^{14} cm^{-2} for ^{12}C and ^{1}H. The SIMS analysis (Fig. S8d) reveals carbon and hydrogen densities of 8.87×10^{18} cm^{-3} and 7.95×10^{18} cm^{-3}, respectively, in the middle of the device layer.

For sample A and B measured in the main text, the implantation fluences are 5×10^{13} cm^{-2} and 7×10^{12} cm^{-2}, respectively. We use the PLE spectra measured when the cavity is far-detuned from multiple devices to estimate the T center density. Due to spectral congestion, the T center peaks near the center of the inhomogeneous distribution are not well resolved. We turn to use the PLE spectrum area to estimate T center density, where we calculate the ratio between the area under the full PLE spectrum and the average area under a well isolated T center peak at the tail of the inhomogeneous distribution. We estimate an average number of ∼100 and ∼30 T centers per device for sample A and B, respectively. These amounts of T centers correspond to a T center density of $\sim 1 \times 10^{12}$ cm^{-3} and $\sim 3 \times 10^{11}$ cm^{-3} for sample A and B, respectively, assuming the majority of the observed T centers are in the taper waveguide region. Using a cavity volume where single T centers can obtain appreciable enhancement (i.e. with simulated Purcell factor $P > 0.1\ P_{\mathrm{max}}$), and the above T center densities, we estimate around 0.082 and 0.025 T centers per cavity for devices in sample A and sample B, respectively. Further optimization of the ion implantation fluence ratio (^{12}C:^{1}H) and the thermal treatment are underway to improve the T center generation yield.

3.2 Nuclear spin density analysis

The nuclear spin bath around the single T center can create magnetic noises and cause dephasing. Here, we analyze potential atomic species with non-zero nuclear spins. To gauge the average distance between neighbouring nuclear spins of the same atomic species, we utilize the following formalism to calculate. We define a probability distribution $w(r)$ to describe the probability of finding a neighbouring nuclear spin within distance r, which satisfies,

$$w(r)dr = \left(1 - \int_0^r w(r')dr'\right)(4\pi r^2 dr)\rho, \tag{S2}$$

where ρ is the nuclear spin volume density. Solving Eq. S2 will lead to a probability distribution function,

$$w(r) = 4\pi\rho r^2 \exp\left(-\frac{4\pi\rho}{3}r^3\right). \tag{S3}$$

The average distance between the same-species nuclear spins (d_{nn}) is then derived as,

$$d_{nn} = \int_0^\infty w(r)rdr = \Gamma(4/3)\left(\frac{4\pi\rho}{3}\right)^{-\frac{1}{3}} \approx 0.554\rho^{-\frac{1}{3}}, \tag{S4}$$

where $\Gamma(z) = \int_0^\infty t^{z-1}e^{-t}dt$ is the Gamma function.

The table below shows major nuclear spin species inside the sample B (C:H = 1:1, 7×10^{12} cm^{-2} implantation fluence). As a reference to the density shown below, the silicon lattice has an atomic density of 5.0×10^{22} cm^{-3}. The boron density is estimated using the SOI device layer resistivity [14].

	^{29}Si (I=1/2)	^{1}H (I=1/2)	^{13}C (I=1/2)	^{11}B (I=−3/2)	^{10}B (I=3)
Density (cm^{-3})	2.34×10^{21}	5.56×10^{17}	3.18×10^{15}	1.07×10^{13}	2.61×10^{12}
Average separation (nm)	0.42	6.7	37.7	251.4	402.4

The major nuclear spin species is ^{29}Si, which can be isotopically purified to reach ∼ ppm level, therefore lowering the density of ^{29}Si to $\sim 10^{16}$ cm^{-3}. As hydrogen is part of the T center, its density can't be decreased without compromising the T center density, which necessitates trade off between them. With a proper density, environmental hydrogen can be utilized as extra nuclear spin quantum memories [15].

4 Waveguide-coupled T centers

With the cavity far-detuned from the scan range, we probe the T centers that are most likely in the taper waveguide region. We note that the spectrum (Fig. 2c of the main text) measured in this case can also include contributions from T centers located in the GC region, but with less quantity as the volume of the GC is ∼10 times smaller than that of the taper waveguide. Figure S9a and S9b summarize the fluorescence lifetime and linewidth from resolvable T center peaks across different devices from sample A and B. The statistical analysis reveals an average lifetime of $\tau_{\text{exc}} = 836.8 \pm 57.3$ ns and FWHM linewidth of $\Gamma = 2.40 \pm 0.86$ GHz.

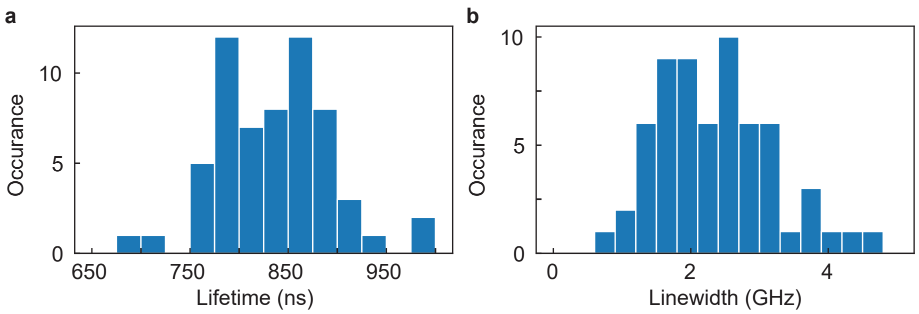

Fig. S9. **Statistics of waveguide-coupled T centers. a.** Excited-state lifetime. **b.** FWHM linewidth.

The average τ_{exc} is slightly shorter than the bulk lifetime of $1/\Gamma_0 = 940$ ns [5], which may come from a finite Purcell enhancement due to the waveguide and/or more unwanted nonradiative decays in the nanophotonic structure

compared with the bulk silicon. The linewidth distribution is similar to previously reported measurements [16]. We note that a large power ($P_{\rm in} \sim \mu$W) is used when measuring the PLE spectrum for waveguide-coupled T centers, therefore power broadening is expected in the measured Γ. The observed cavity-coupled T center, unfortunately, has a linewidth at the higher end of this distribution. Further investigation is needed to verify whether single T centers inside the cavity have a systematically larger linewidth compared with those in the SOI waveguides or bulk silicon.

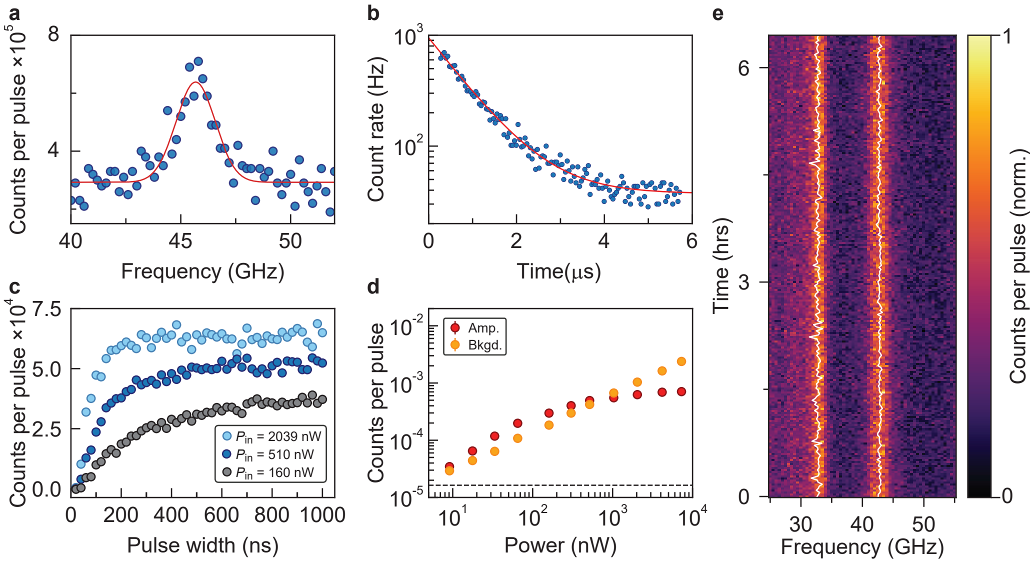

Fig. S10. **Waveguide-coupled T center characterizations.** **a.** PLE spectrum of a waveguide-coupled T center at $P_{\rm in} = 9.12$ nW. Red line is the Gaussian fitting. **b.** Time-resolved fluorescence decay for the same T center at $P_{\rm in} = 302.68$ nW. **c.** Optical Rabi measurement at different powers. **d.** Saturation curve with an excitation pulse width of 600 ns. The dashed black line is the contribution from the detector dark counts. Here the collection window misses the first 300 ns of the fluorescence decay. **e.** Spectral diffusion of two waveguide-coupled T centers at an excitation power $P_{\rm in} = 60.93$ nW. White solid line shows the Gaussian fitted peak center at each iteration (with a duration of 102.5 seconds). The T center occasionally shows spectral jumps, causing the peak position difference between two measurements of the same T center located at 45.67 GHz in panel **a** and 42.78 GHz in panel **e**.

We investigate the optical properties of a specific waveguide-coupled T center located at 45.67 GHz in sample B, which has a low-power linewidth $\Gamma = 2.10 \pm 0.18$ GHz (Fig. S10a) and a fluorescence lifetime of $\tau_{\rm exc} = 838.2 \pm 8.1$ ns (Fig. S10b). Due to the fast dephasing, this waveguide-coupled T center is incoherently driven to saturation without any Rabi oscillations (Fig. S10c). The emission of this waveguide-coupled T center shows typical saturation behavior (Fig. S10d) with a saturated count level of $C_p^{\rm sat} = 7 \times 10^{-4}$ photon counts per pulse. Using a similar procedure discussed in Section 1.3, we can define a system collection efficiency for a waveguide-coupled T center as $\eta_{\rm sys,wg} = \eta_{\rm wg}\eta_{\rm col}\eta_{\rm path}\eta_{\rm SNSPD} = 0.0785 \times \eta_{\rm wg}$, where $\eta_{\rm wg}$ is the efficiency for the T center emission being coupled to the waveguide mode. The $\eta_{\rm col} = 0.142$ is the fraction of the T center ZPL and PSB outcoupled by the GC, estimated using the T center DW factor and PSB spectrum under resonant PL excitation reported in literature [5]. The saturation count level can thus be calculated as $C_p^{\rm sat} = 0.5e^{-t_0/\tau_{\rm exc}}\eta_{\rm QE}\eta_{\rm sys,wg}$, which let us extract the waveguide efficiency $2.55\% \leq \eta_{\rm wg} \leq 10.90\%$. Similarly as in Fig. 3e of the main text, we monitor the stability of two waveguide-coupled T centers for up to 6 hours (Fig. S10e), revealing a spectrum-center distribution of 32.73 ± 0.31 GHz and 42.78 ± 0.18 GHz for the two T centers, respectively.

5 Cavity-coupled single T center

5.1 Optical Rabi

Due to the large dephasing rate, we have not been able to drive coherent optical Rabi oscillations of the cavity-coupled single T center (Fig. S11) up to an excitation power of $P_{\text{in}} = 163.43$ nW, which corresponds to a Rabi frequency $\Omega = 2\pi \times 586$ MHz. We use a pulse width of 900 ns to ensure incoherent driving for the cavity-coupled single T center in different measurements.

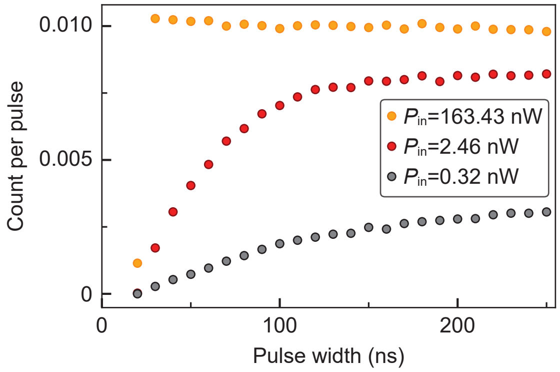

Fig. S11. **Optical Rabi measurement for the cavity-coupled single T center.** The curve is measured when the cavity is on resonance. The counts per pulse is background corrected.

5.2 Second-order autocorrelation $g^{(2)}(0)$ analysis

Higher $g^{(2)}(0)$ is observed at increasing excitation powers during the saturation measurements (Fig. 3d of the main text). The emission from waveguide-coupled T centers (e.g., those from the taper waveguide and the GC) contributes to the accidental $g^{(2)}(0)$ coincidences, which leads to a dependence of $g^{(2)}(0)$ on the signal-to-noise ratio (SNR, A). We note that the detector dark counts (3 Hz) have much less influence on the A. The $g^{(2)}(0)$ can be described as [17],

$$g^{(2)}(0) = \frac{2A+1}{(A+1)^2}. \tag{S5}$$

The SNR, A, is first extracted using the Gaussian-fitted amplitude and background for each PLE spectrum. Predicted $g^{(2)}(0)$ based on Eq. S5 (red dashed line in Fig. S12) surpasses the experimental results, which is due to the inaccurate estimation of the PLE spectrum background. To better estimate the A, the background is obtained by using the average spectrum intensity at a region away from the T center peak. The updated $g^{(2)}(0)$ prediction (orange dashed line in Fig. S12) shows a good match with the data, confirming the $g^{(2)}(0)$ is limited by the SNR.

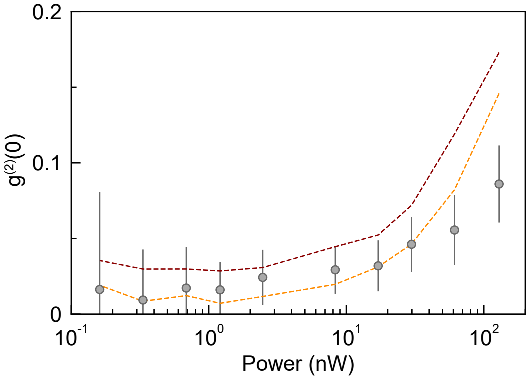

Fig. S12. **Second-order autocorrelation.** $g^{(2)}(0)$ as a function of P_{in} for the cavity-coupled single T center at $\Delta_{ac} = 0$. Dashed lines show the SNR-limited $g^{(2)}(0)$ calculated by Eq. S5 with the SNR estimated using a background from the Gaussian fitting (red) and from the average spectrum intensity at a region away from the T center peak (orange).

5.3 Bunching behavior of the autocorrelation

In this section, we discuss the bunching features in the autocorrelation measurements of the cavity-coupled single T center. A typical $g^2(n)$ with bunching behavior is shown in Fig. S13a. We measure the autocorrelation under different experimental conditions (P_{in}, pulse width, Δ_{ac}) to investigate factors that may affect the bunching feature (Fig. S13b−e). Qualitatively, the bunching is less pronounced when the cavity-coupled single T center is driven stronger (e.g., under higher power, wider pulse width, and smaller detuning).

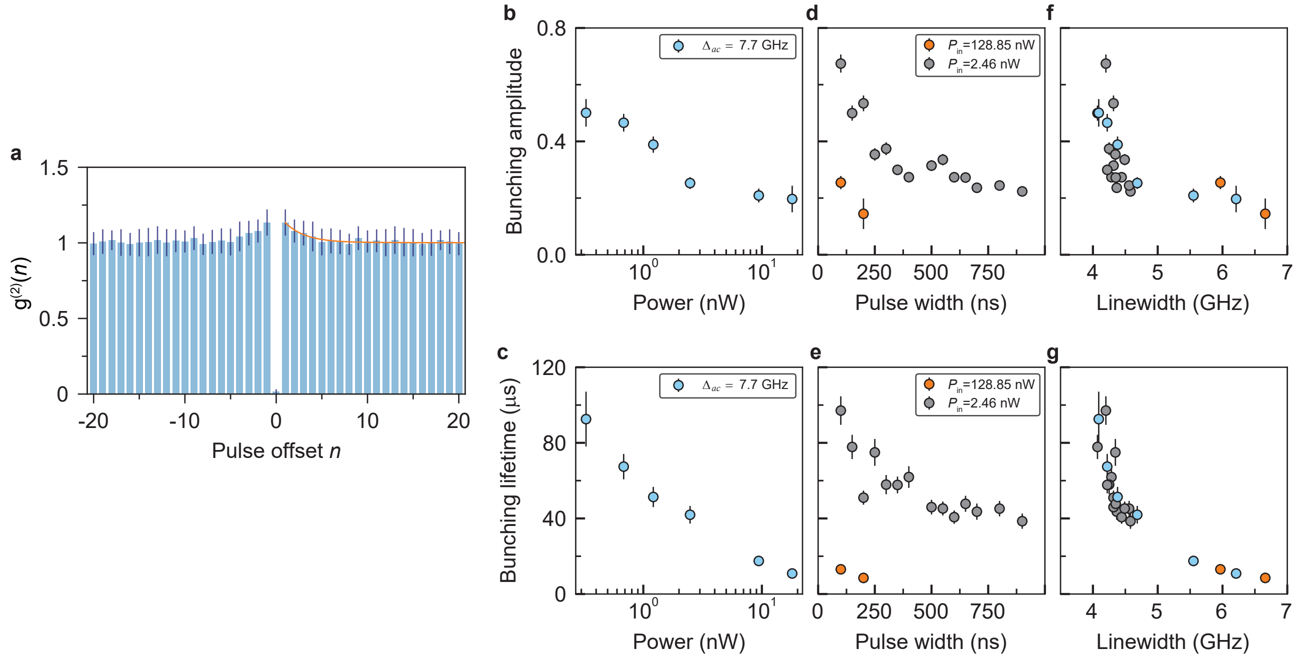

Fig. S13. **Autocorrelation bunching behavior. a.** A representative $g^2(n)$ plot that shows bunching feature with $\Delta_{ac} = 7.7$ GHz, under an excitation power of $P_{\text{in}} = 9.37$ nW with an excitation pulse width of 900 ns. The single-exponential decay (orange line) reveals a bunching lifetime of 17.5 ± 2.3 μs. Here the bunching lifetime is presented as nT_{seq}, where $T_{\text{seq}} = 8$ μs is the length of each pulse sequence. Panels **b** and **c** show the bunching amplitude and lifetime under different input powers at $\Delta_{ac} = 7.7$ GHz with a pulse width of 900 ns. Panels **d** and **e** show the bunching amplitude and lifetime under two excitation powers at different pulse widths ($\Delta_{ac} = -7.7$ GHz). Panels **f** and **g** show the summarized bunching amplitude and lifetime results from panel **b** − **e**, but now using the corresponding PLE spectrum linewidth as the x-axis.

The bunching in the autocorrelation measurements for a single T center at zero magnetic field can be the result of transient occupation of a dark state (e.g., a shelving state or a charge state) or spectral diffusion [18]. Similar bunching dynamics have been reported in single G centers in silicon due to a shelving sate [19]. However, such a shelving mechanism has not been demonstrated for single T centers to the best of our knowledge. The bound exciton excited states (e.g., TX_1, TX_2...) are likely having very short lifetimes due to the fast phonon relaxation. As the bunching decay lifetimes are at tens of μs time scales ($\gg$ fluorescence decay), we conclude that they can't originate from the bound exciton excited states. Charge state seems unlikely for the cavity-coupled single T center as no fluorescence intermittency is observed (Fig. 3e of the main text), and the count level keeps at saturation under a wide range of high powers (Fig. 3c of the main text).

The above analysis leads us to believe the spectral diffusion is the main origin of the observed bunching feature. The cavity-coupled single T center indeed has a large spectral diffusion as shown in Section 6. When summarizing all bunching lifetimes and amplitudes against PLE spectrum linewidth (Fig. S13f−g), we observe strong dependence unexpectedly. Further investigations are needed to illustrate the specific mechanisms involved here. Several noise sources can generate spectral diffusion, including the magnetic field noises due to the nuclear spin bath of ^{29}Si and potential electrical field noises from silicon surface charge states or other impurities existing in the silicon.

5.4 Magnetic field test

The electronic structure of a single T center consists of a ground state formed by an isotropic electron spin (g-factor $g_e = 2.005$), and an excited state formed by an anisotropic hole spin [5]. Depending on the T center orientation with respect to the applied magnetic field B, the anisotropic hole spin has a g-factor (g_h) ranging from 1.609 to 3.457

when the B field is along the silicon $\langle 110\rangle$ direction. For B field along the $\langle 100\rangle$ direction, the g_h values for the twelve possible T center orientational subsets reduce to $g_h = \{0.91, 2.55\}$ with multiplicities of 4 and 8, respectively [20].

We apply a magnetic field along the silicon [100] direction up to 300 mT aiming to split the cavity-coupled single T center ZPL transition. To avoid optical pumping that may polarize the electron spin [5], we use the EOM to generate RF sidebands ($f_0 \pm f_{\mathrm{RF}}$) of the laser carrier (f_0) and drive the two spin conserving transitions simultaneously (transitions A and B in Fig. S14a). By scanning the f_{RF}, we expect to locate a fluorescence peak when $f_{\mathrm{RF}} = \frac{1}{2}|\Delta_g|\mu_B B$, where μ_B is the Bohr magneton and $|\Delta_g| = |g_e - g_h|$ is the difference of the excited- and ground-state g-factors. We note that we have not been able to observe such a Zeeman splitting, which may be due to the insufficient splitting compared with the broad linewidth of the cavity-coupled single T center.

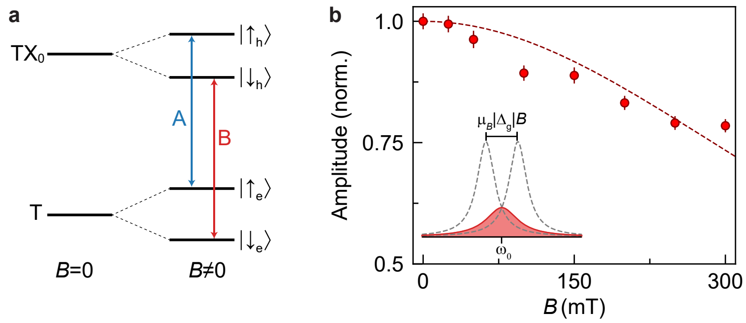

Fig. S14. **Magnetic field test.** **a.** Schematic of T center electronic structure. With $B \neq 0$, the doublet ground and TX_0 states will be split. **b.** PLE peak intensity decrease of the cavity-coupled T center ($\Delta_{ac} = 0$) at increasing magnetic field along silicon [100] direction. The experimental data is fitted by Eq. S6 (dashed line).

We then turn to the single-tone laser carrier scan. The optical pumping will cause electron spin polarization [5], which reduces the PLE signal. However, the broad single T center linewidth enables the single frequency excitation to address both spin conserving transitions in their spectral overlap (inset in Fig. S14b). As the separation between A and B transitions increases at higher B field, the probability of driving both transitions decreases, which leads to spin polarization and the lowering of the PLE signal. We note that the T center position and its fluorescence lifetime remain the same across the whole magnetic field test, suggesting minimal influence from the coupling with the cavity.

Assuming that the optical linewidth Γ for transitions A and B are both equal to the linewidth for the cavity-coupled single T center at zero field ($\Gamma = 3.83$ GHz under $P_{\mathrm{in}} = 0.08$ nW), we can model the amplitude of the field-dependent PLE spectrum using the following equation [20],

$$I(B) = \frac{\Gamma^2}{\Gamma^2 + (|\Delta_g|\mu_B B)^2}. \tag{S6}$$

For simplicity, we have neglected the minor contributions from the spin-nonconserving transitions. By fitting the field dependent PLE spectrum amplitudes (Fig. S14b), we extract $|\Delta_g| = 0.55 \pm 0.04$, which is in close agreement with the theoretical prediction [20].

5.5 Thermal broadening

The cavity-coupled single T center emission line can also be thermally broadened due to activation of the TX_0-TX_1 transition [5]. To estimate the thermal broadening, we measure the temperature-dependent linewidth for the single T center (Fig. S15) and fit the results using,

$$\Gamma(T) = P_0 + \frac{P_{\mathrm{T}}}{e^{Ea/k_B T} - 1}, \tag{S7}$$

where P_0 is the linewidth at $T = 0$ K, P_{T} is the thermal broadening coefficient, and E_a is the activation energy. The fitted $E_a = 1.35 \pm 0.02$ meV is close to the reported 1.76 meV TX_0-TX_1 state splitting [5]. The comparison between P_0 and the measured linewidth of the cavity-coupled single T center at $T = 3.4$ K suggests a thermal broadening value of $\Gamma_{\mathrm{th}} \sim 0.1$ GHz.

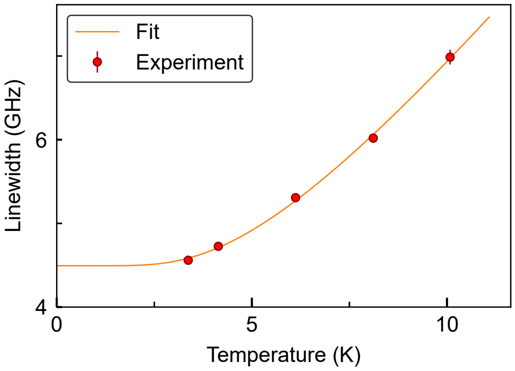

Fig. S15. **Thermal broadening**. The measured temperature-dependent linewidth is fitted using Eq. S7. The fitted parameters are E_a=1.35 ± 0.02 meV, P_{T}=9.21 GHz, P_0=4.49 GHz.

5.6 Lifetime analysis

Due to the spectral overlap between the cavity-coupled single T center with the inhomogeneous distribution of the waveguide-coupled T centers, background T center emission is expected while measuring the fluorescence decay of the cavity-coupled T center (Fig. 4a of the main text). Here we compare the fitting results of the decay data using single- and bi-exponential functions (Fig. S16a). The single-exponential fitting reveals a fluorescence decay lifetime $\tau_{\mathrm{exc}} = 144.3 \pm 0.2$ ns, yet showing a large deviation at longer time scales. With bi-exponential fitting, the dominant decay has a fitted lifetime of $\tau_{\mathrm{exc}} = 136.4 \pm 0.6$ ns with an amplitude weight of 97.0%. For the cavity-coupled single T center, the measured lifetime has no dependence on the excitation power (Fig. S16b), which rules out any forms of stimulated emission process.

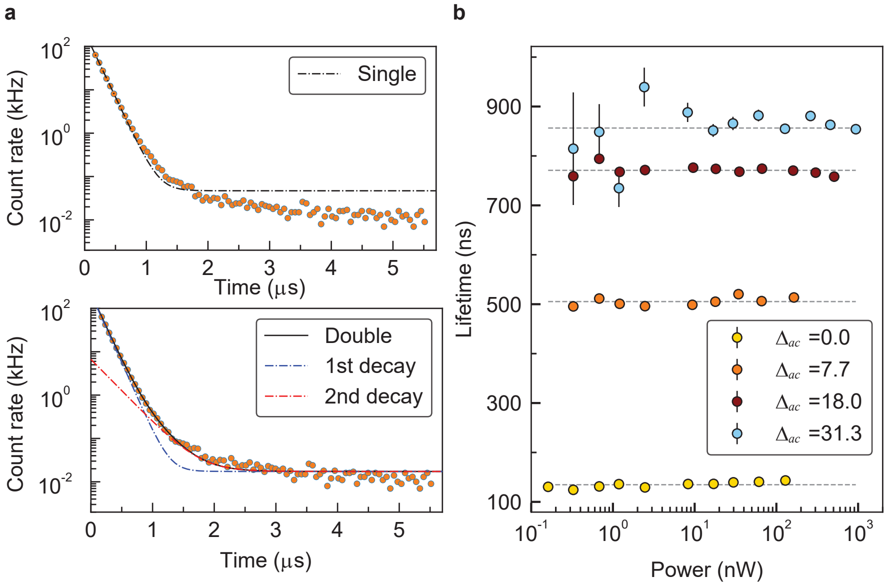

Fig. S16. **Fluorescence decay lifetime analysis. a.** Single- and bi-exponential fitting for the cavity-coupled single T center fluorescence decay at $\Delta_{ac} = 0$ (Fig. 4a of the main text). Single-exponential fitting (upper panel) reveals $\tau_1 = 144.3$ ns, with a count-rate amplitude $C^1_{\mathrm{max}} = 213.4$ kHz. Bi-exponential fitting (lower panel) reveals $\tau_1 = 136.4$ ns and $\tau_2 = 298.1$ ns, with count-rate amplitudes $C^1_{\mathrm{max}} = 217.1$ kHz and $C^2_{\mathrm{max}} = 6.7$ kHz, respectively. **b.** Fluorescence lifetime of the cavity-coupled single T center under different excitation powers at certain detuning Δ_{ac}. For the $\Delta_{ac} = 0$ case, we perform bi-exponential fitting and use the lifetime for the major decay. The average lifetime for each detuning is 134.7, 505.3, 770.8 and 856.5 ns.

6 Numerical modeling of the coupled system

6.1 Lindblad master equation

The dynamics of the coupled system for a single T center in a cavity can be well described by the Jaynes-Cummings Hamiltonian H_{JC} of the form,

$$H_{\mathrm{JC}}/\hbar = H_0 + H_d, \tag{S8}$$

$$H_0 = \Delta_a \sigma_+ \sigma_- + \Delta_c a^\dagger a + g(\sigma_+ a + \sigma_- a^\dagger), H_d = \frac{\Omega}{2}(\sigma_+ + \sigma_-), \tag{S9}$$

which assumes rotating wave approximation and is in the rotating frame of the laser field (ω_L). Here $\Delta_a = \omega_a - \omega_L$ and $\Delta_c = \omega_c - \omega_L$ are, respectively, the detunings of the laser from the T center transition ω_a and from the cavity resonance ω_c, g is the coupling strength between the single T center and the single cavity mode, Ω is the optical Rabi frequency, a and σ_- are the annihilation operators for the cavity mode and the T center optical transition, respectively. The optical Rabi frequency $\Omega = 2g\sqrt{N_{\mathrm{ph}}}$, where $2g$ is the single photon Rabi frequency and N_{ph} is the average intracavity photon number. The N_{ph} is related to the input power P_{in} by the following equation [21],

$$N_{\mathrm{ph}} = 4\frac{\eta_{\mathrm{cav}}/\kappa}{1+(2\Delta_c/\kappa)^2}\frac{P_{\mathrm{in}}}{\hbar\omega_L}, \tag{S10}$$

where κ is the cavity linewidth. We note the equivalence between semiclassical drive $H_d = \frac{\Omega}{2}(\sigma_+ + \sigma_-)$ and coherent drive $H_d = \sqrt{\frac{\kappa_{\mathrm{wg}} P_{\mathrm{in}}}{\hbar\omega_L}}(a^\dagger + a)$, given a sufficiently large photon Fock state space. To analyze the open quantum system, we utilize the Lindblad master equation in QuTiP [22] to describe the time evolution of the light-matter interaction,

$$\frac{d}{dt}\rho = \frac{-i}{\hbar}[H_{\mathrm{JC}}(t), \rho] + \sum_i C_i \rho C_i^\dagger - \frac{1}{2}\{C_i^\dagger C_i, \rho\} \equiv \mathcal{L}\rho. \tag{S11}$$

The jump operators C_i describe different loss mechanisms. We define major loss channels as $\sqrt{\kappa}a$, $\sqrt{\Gamma_0}\sigma_-$, and $\sqrt{\Gamma_{\mathrm{d}}/2}\sigma_z$, which respectively represent cavity decay, T center spontaneous emission, and pure dephasing with a rate of Γ_{d}. To simulate the pulsed excitation, we implement a time-dependent Hamiltonian $H(t)$, which consists of $H_0 + H_d$ during the excitation and H_0 when the excitation is off. To take into account the possible spectral diffusion (Γ_{sd}) of the single T center, we perform a weighted average of the simulation outcomes assuming Δ_a takes a range of values with each weight determined by a Gaussian distribution with a FWHM linewdith of $2\Gamma_{\mathrm{sd}}$.

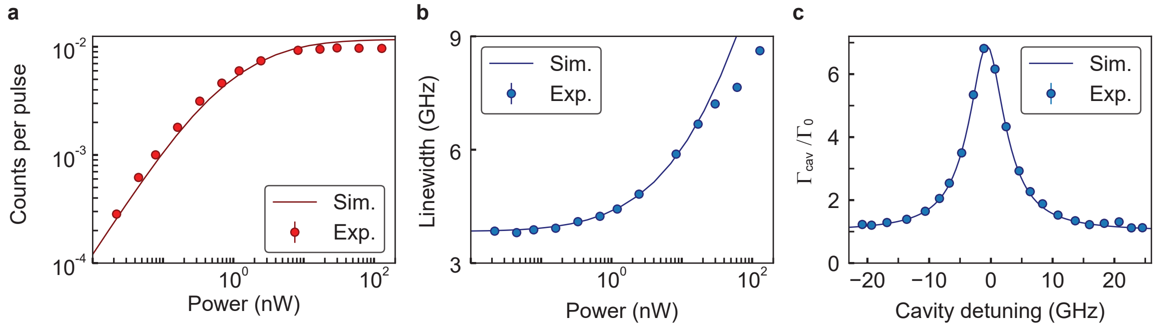

Fig. S17. **Lindbladian calculation results.** Global numerical fitting of the saturation (**a**), linewidth (**b**) and detuning-dependent fluorescence decay (**c**) data (reproduced from Fig. 3c, Fig. 3d, and Fig. 4b of the main text) by solving the Lindblad master equation, using g, Γ_{d} and Γ_{sd} as the fitting parameters.

We extract the g, Γ_{d} and Γ_{sd} by fitting counts saturation, power-dependent linewidth, and detuning-dependent fluorescence decay data globally using the SciPy built-in function Basin-hopping and Nelder-Mead method [23], which reveals (g, Γ_{d}, Γ_{sd}) $=2\pi\times$(42.4 MHz, 0.65 GHz, 1.69 GHz). The global fitting can match with the different measurement data simultaneously (Fig. S17). The characteristic linewdith of $\tilde{\kappa}$ has contributions from the cavity linewidth κ, the dephasing Γ_{d}, and the spectral diffusion Γ_{sd}. In a hypothetical scenario where Γ_{sd} is negligible, we can have an analytical formula for $\tilde{\kappa} \approx \kappa + 2\Gamma_{\mathrm{d}}$ derived from the numerical model mentioned above.

6.2 Cavity-coupled spectrum

As the cavity resonance is tuned across the cavity-coupled single T center optical transition, we observe modulation of the T center spectrum depending on the cavity detuning $\Delta_{ac} = \omega_c - \omega_a$ (Fig. S18a). This is mainly attributed to the dependence of the optical excitation on the Δ_c, which results from the comparable linewidth of the single T center and the cavity ($\Gamma \sim \kappa$). The driving strength across the T center linewidth varies because the average intracavity photon number N_{ph} depends on the ω_L, which can be seen by rewriting Eq. S10 as,

$$N_{\text{ph}} = 4\frac{\eta_{\text{cav}}/\kappa}{1+[2(\Delta_{ac}+\omega_a-\omega_L)/\kappa]^2}\frac{P_{\text{in}}}{\hbar\omega_L}. \tag{S12}$$

For a specific cavity detuning Δ_{ac}, when ω_L is scanned (with a typical range $\sim 5\Gamma$) to obtain the single T center PLE spectrum, N_{ph} will be modulated strongly. In a hypothetical regime where $\Gamma \ll \kappa$, this effect will disappear.

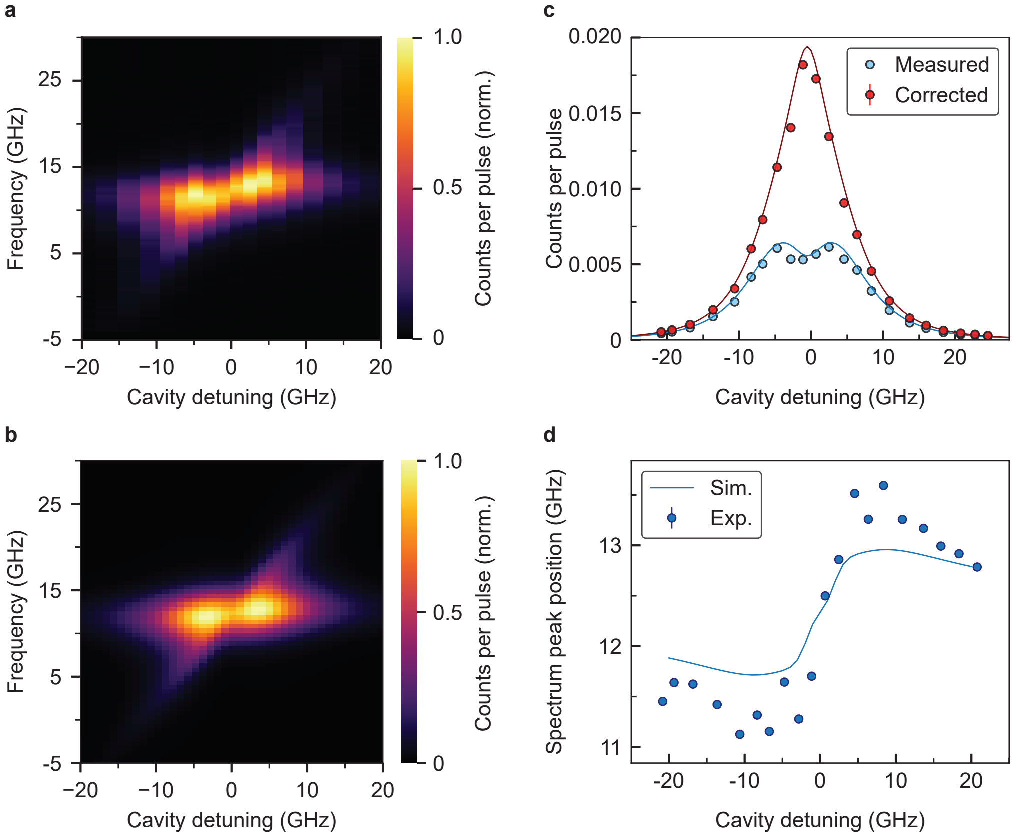

Fig. S18. **Cavity-coupled single T center spectrum. a.** Measured 2D spectrum map for the cavity-coupled single T center at different Δ_{ac} under an excitation power $P_{\text{in}} = 1.21$ nW. **b.** Simulated 2D spectrum map based on the numerical calculations. **c.** Measured and corrected counts at the T center optical transition (ω_a) at different Δ_{ac}. The correction factor is $e^{t_0/\tau_{\text{exc}}}$. The solid lines are the results from the numerical calculations. **d.** Extracted PLE spectrum peak positions at different Δ_{ac}. The blue line represents the numerical calculation results.

We turn to the numerical calculations to verify the observed 2D spectrum map (Fig. S18a). We use the fitted (g, Γ_{d}, Γ_{sd}) determined in Section 6.1 for solving the Lindblad master equation to predict the single T center emission spectrum at different cavity detunings. The predicted 2D spectrum map (Fig. S18b) match well with the measurements. The lower count level at $\Delta_{ac} = 0$ is due to the $t_0 = 170$ ns time window where the fluorescence cannot be collected (see Section 1.3), which affects more at zero detuning as the T center has a shorter fluorescence lifetime. The numerical calculations also well capture the raw and corrected count level (Fig. S18c) at the T center optical transition ($\omega_a = 12.34$ GHz), as well as the evolution of the peak position for the cavity-coupled PLE spectrum at different cavity detunings (Fig. S18d).

References

[1] Mosor, S. *et al.* Scanning a photonic crystal slab nanocavity by condensation of xenon. *Appl. Phys. Lett.* **87**, 141105 (2005).

[2] Chan, J., Eichenfield, M., Camacho, R. & Painter, O. Optical and mechanical design of a "zipper" photonic crystal optomechanical cavity. *Opt. Express* **17**, 3802–3817 (2009).

[3] Johnson, S. G. & Joannopoulos, J. D. Block-iterative frequency-domain methods for Maxwell's equations in a planewave basis. *Opt. Express* **8**, 173–190 (2001).

[4] Oskooi, A. F. *et al.* MEEP: A flexible free-software package for electromagnetic simulations by the FDTD method. *Comput. Phys. Commun.* **181**, 687–702 (2010).

[5] Bergeron, L. *et al.* Silicon-integrated telecommunications photon-spin interface. *PRX Quantum* **1**, 020301 (2020).

[6] Dung, H. T., Buhmann, S. Y. & Welsch, D.-G. Local-field correction to the spontaneous decay rate of atoms embedded in bodies of finite size. *Phys. Rev. A* **74**, 023803 (2006).

[7] Liu, L., Pu, M., Yvind, K. & Hvam, J. M. High-efficiency, large-bandwidth silicon-on-insulator grating coupler based on a fully-etched photonic crystal structure. *Appl. Phys. Lett.* **96** (2010).

[8] Ding, Y., Ou, H. & Peucheret, C. Ultrahigh-efficiency apodized grating coupler using fully etched photonic crystals. *Opt. Lett.* **38**, 2732–2734 (2013).

[9] Halir, R. *et al.* Waveguide sub-wavelength structures: a review of principles and applications. *Laser & Photon. Rev.* **9**, 25–49 (2015).

[10] Halir, R. *et al.* Waveguide grating coupler with subwavelength microstructures. *Opt. Lett.* **34**, 1408–1410 (2009).

[11] Chen, S. *et al.* Hybrid microwave-optical scanning probe for addressing solid-state spins in nanophotonic cavities. *Opt. Express* **29**, 4902–4911 (2021).

[12] Ziegler, J. F., Ziegler, M. & Biersack, J. SRIM – The stopping and range of ions in matter (2010). *Nucl. Instrum. Methods Phys. Res. B: Beam Interact. Mater. At.* **268**, 1818–1823 (2010).

[13] Cho, K. *et al.* Channeling effect for low energy ion implantation in Si. *Nucl. Instrum. Methods Phys. Res. B: Beam Interact. Mater. At.* **7-8**, 265–272 (1985).

[14] Thurber, W., Mattis, R., Liu, Y. & Filliben, J. Resistivity-dopant density relationship for boron-doped silicon. *J. Electrochem. Soc.* **127**, 2291 (1980).

[15] Uysal, M. T. *et al.* Coherent control of a nuclear spin via interactions with a rare-earth ion in the solid state. *PRX Quantum* **4**, 010323 (2023).

[16] Higginbottom, D. B. *et al.* Optical observation of single spins in silicon. *Nature* **607**, 266–270 (2022).

[17] Dibos, A., Raha, M., Phenicie, C. & Thompson, J. D. Atomic source of single photons in the telecom band. *Phys. Rev. Lett.* **120**, 243601 (2018).

[18] Sallen, G. *et al.* Subnanosecond spectral diffusion measurement using photon correlation. *Nat. Photon.* **4**, 696–699 (2010).

[19] Redjem, W. *et al.* Single artificial atoms in silicon emitting at telecom wavelengths. *Nat. Electron.* **3**, 738–743 (2020).

[20] MacQuarrie, E. R. *et al.* Generating T centres in photonic silicon-on-insulator material by ion implantation. *New J. Phys.* **23**, 103008 (2021).

[21] Tiecke, T. *et al.* Nanophotonic quantum phase switch with a single atom. *Nature* **508**, 241–244 (2014).

[22] Johansson, J. R., Nation, P. D. & Nori, F. QuTiP: An open-source Python framework for the dynamics of open quantum systems. *Comput. Phys. Commun.* **183**, 1760–1772 (2012).

[23] Virtanen, P. *et al.* SciPy 1.0: fundamental algorithms for scientific computing in Python. *Nat. Methods* **17**, 261–272 (2020).
